# Orofacial Pathologic Lesions in Children and Adolescents: A Clinicopathological Study in Southern Iran

**Published:** 2014-06

**Authors:** Zohreh Jaafari Ashkavandi, Zahra Ahmadi Sheshdeh, Fereshteh Kamali

**Affiliations:** Department of Oral and Maxillofacial Pathology, School of Dentistry, Shiraz University of Medical Science, Shiraz, Iran

**Keywords:** Pathologic Lesion; Oral Pathology; Maxillofacial Malignancy

## Abstract

***Objective:*** Oral and maxillofacial lesions vary regarding their clinical presentation in different populations. Until now, oral and maxillofacial lesions in Iranian children and adolescents have not been studied. The aim of this study was to determine the type and distribution of biopsied oral lesions among children and adolescents in Southern Iran.

***Methods:*** All the patients referred to the pathology department of Shiraz Faculty of Dentistry from 1991-2009 were enrolled in this retrospective, case-series study. The information regarding the patients’ age, gender as well as the histopathologic type and anatomic location of the biopsied oral lesions in patients under 18 years was collected from patients’ medical documents and were analyzed by SPSS version 11.

***Findings*** ***:*** Out of 2984 patients, 576 (19.3 %) cases were children and adolescents under 18 years. The most prevalent category was soft tissue lesions (45.5 %). The most common lesion was peripheral giant cell granuloma (15.6%) followed by dentigerous cyst (14.2%) and pyogenic granuloma (11.3%). Gingiva was the most common affected site. Male to female ratio was 1.2.

***Conclusion:*** Our results revealed that near 20% of orofacial lesions occur in children and adolescents with rather equal male to female ratio. The majority of lesions were soft tissue diseases with a reactive nature. Unlike other studies we had higher rates of soft tissue lesions. These data can help dentists and surgeons for more accurate management of their patients.

## Discussion

This study evaluated the oral pathologic lesions in a group of children and adolescents and found that these patients comprise 19.2 % of total cases.

 This rate was higher than the rate reported by the previous researchers in USA (7%)^[^^9^^]^, U.K (8.2%)^[^^10^^]^, China (5.5%)^[^^3^^] ^andThailand (15%)^[^^7^^]^ but Lawoyin et al^[^^12^^]^ have reported a prevalence of25% for the pathologic lesions in Nigerian children and adolescents under 17 years. It should be noted that the age range of the patients have been different in these studies (under 15 to under 18 years old)^[^^6^^,^^7^^,^^9^^,^^10^^]^.

**Table 1:** Soft tissue lesions

	**Number**			**% of** ** total**	**Male/Female ** **ratio**	**Most common** ** location**
	**children**	**adolescents**				
**PGCG**	76		16	35/1	2	Gingiva
**Pyogenic Granuluma**	33		34	24/8	1	Gingiva
**Peripheral Ossifying Fibroma**	9		32	16/4	1/1	Gingiva
**Irritation Fibroma**	7		15	8/4	0/6	Gingiva
**Fibroepithelial Polyp**	8		9	6/5	0/5	Gingiva
**Hemangioma**	4		6	3/8	4	Buccal mucosa
**Fibrous Histiocytoma**	2		3	2	1/5	Gingiva
**Giant cell Fibroma**	2		1	1/2	0:3	Gingiva
**Neurofibroma**	3		-	1/2	0:3	Buccal mucosa
**Desmoplastic Fibroma**	2		-	0/8	1	Gingiva
**Total **	146		116	-	-	

**Table 2:** Odontogenic cysts and tumors

**Lesions**	**Number**		**% of ** **total**	**Male/Female** ** ratio**	**Most common** ** location**
	**adolescents**	**children **			
**Dentigerus Cyst**	53	29	71/4	2/03	Mand
**KOT**	6	20	22/6	1/16	Max = Mand
**Eruption Cyst**	6	0	5/2	6:0	Mand
**Calcifying Odontogenic Cyst**	0	1	0/8	0:1	Max
**Odontoma**	4	5	27/3	2	Max
**Ameloblastoma**	1	8	27/3	1/25	Mand
**AOT**	-	5	15/2	0/25	Mand
**Odontogenic Fibroma**	1	3	12/2	1	Max
**Cementoblastoma**	-	3	9	0/5	Mand
**Ameloblastic Fibroma**	2	-	6	0:2	Max = Mand
**Myxoma**	1	-	3	1:0	Mand
**Total**	74	74			

KOT: Keratocystic odontogenic tumor; AOT: Adenomatoid odontogenic cyst

Evaluation of clinical characteristics of pathologic lesions can help clinicians for a better clinical management of the any pathologic lesion, demographic data of the patient should be considered. These data are influenced by geographic and ethnic factors, so epidemiologic studies in any population seem to be important.

 The frequency of oral lesions was almost equal in male and female groups. This finding was in agreement with other studies that have shown M:F ratio to be 1-1.2^[^^6^^,^^7^^,^^10^^,^^11^^]^. In the current study, the oral lesions in children and adolescents showed the same incidence. The studies that have evaluated the oral lesions in a long-term have stated that the prevalence of lesions increased with age^[^^6^^,^^9^^,^^10^^,^^11^^]^. In a study that has been done in Thailand, the patients were classified into three groups regarding their dental age: permanent, mixed and deciduous dentition. The authors stated that the prevalence of oral lesions was higher in mixed dentition group^[^^7^^]^. Also, the incidence of oral lesions in children and adolescents was respectively 52% and 48% in Jones et al study^[^^10^^]^ and 48.2% and 51.8% in Chen et al study^[^^6^^]^. It seems that the overall prevalence of oral pathologic disorders was almost equal between children and adolescent groups. Moreover, it should be considered that the prevalence of any specific lesion may be different in the mentioned groups. 

 Regarding the histopathologic type of the subjects, the present study, in line with the previous studies, showed that reactive soft tissue lesions as well as cystic lesions occurred more frequently than other lesions^[^^6^^,^^7^^,^^9^^,^^10^^]^, but one study done on African children and adolescents has shown that neoplasms were more common than inflammatory lesions^[^^12^^]^.

**Table 3:** Salivary gland disease and epithelial pathology and hematologic disorders

**Lesions**	**Number**		**% of total**	**M/F ratio**	**Most common** ** location**
	**children**	**children**			
**Muscocele**	20	25	93/7	1/5	Lower lip &
					Floor of mouth
**Mucoepidermoid Carcinoma**	-	2	4	1	Parotid
**Adenoid Cystic Carcinoma**	1	-	2	1:0	Palate
**Necrotizing Sialometaplasia**	1	-	2	1:0	Palate
**Pleomorphic Adenoma**	1	-	2	0:1	Parotid
**Epithelial Hyperplasia**	3	4	53/5	1/3	Gingiva
**Squamous Papilloma**	2	3	39	0:5	Lip and tongue
**Nevus**	-	1	7/5	0:1	Buccal mucosa
**Lymphoma**	4	1	71/5	5:0	Vestibule
**Eosinophilic granuluma**	-	2	28/5	1	Mand = Max
**Total**	32	38	-	-	-

**Table 4:** Pulp and periapical diseases and bone pathology

**Lesions**	**Number**		**% of total**	**M/F ratio**	**Most common** **location**
	**adolescents**	**children**			
**Radicular cyst**	15	18	91/7	1/3	Max
**Residual cyst**	1	2	8/3	2	Mand
**CGCG**	16	11	45	0/7	Mand
**Fibrous Dysplasia**	2	7	15	1	Max
**Central Ossifying Fibroma**	3	5	13/3	1	Max
**Aneurysmal** ** Bone Cyst**	3	4	11/7	0:7	Mand
**Traumatic Bone Cyst**	0	6	10	1	Mand
**Osteosarcoma**	1	1	3/4	1	Max
**Chondrosarcoma**	0	1	1/6	0:1	Max
**Total**	41	55			

CGCG: central giant cell granuloma

This finding may have been the result of considering reactive lesions (such as ossifying fibroma) as a neoplasm in that study and also, due to the endemic occurrence of Burkitt's lymphoma in African children.

 PGCG was the most common lesion in our study; however, other authors have reported mucocele, dentigerous cyst, radicular cyst, and pyogenic granuloma as the most common oral lesion, respectively in southern Taiwan and Chile^[^^6^^,^^13^^,^^14^^]^, Greece^[^^15^^,^^16^^]^, Thailand^[^^7^^]^ and Brazil^[^^11^^,^^17^^]^. Trauma and poor oral hygiene are the major etiologic factors for the mentioned lesions, except dentigerous cyst. In contrast to our results in children, pyogenic granuloma in adults shows a definitive female predilection, probably because feminine hormones affect the vascular events^[^^8^^,^^18^^]^.

 Odontogenic cysts were the second most common group which constituted 20% of all lesions. This incidence was lower than that of the studies performed in Africa^[^^12^^]^, Thailand^[7]^ and Taiwan^[^^6^^]^ (22-35%), but higher than in Turkey (12%)^[^^19^^]^. In the most studies, dentigerous cyst was the most common cyst^[^^6^^,^^7^^,^^9^^,^^12^^,^^20^^]^. In our study, dentigerous cyst and then odontogenic keratocyst (OKC) were reported more frequently. This finding has been confirmed by other researchers^[^^4^^,^^7^^,^^13^^]^. Odontogenic cysts arise from odontogenic epithelium of tooth germ, and formation of these lesions during development of teeth in the first two decades of life occur frequently^[^^8^^]^.

 Bone lesions were the third most common group of lesions which constituted 10.3% of all. Other studies have been reported this incidence from 3.2 to 4.8%^[^^10^^,^^20^^]^. The present study and all the other studies showed that central giant cell granuloma and fibrous dysplasia were the most common lesions in this group. Respectively, mandible and maxilla were the most frequent sites of involvement, as reported by Lawoyin et al^[^^12^^]^. However, Maya et al reported that both lesions were more frequent in mandible^[^^17^^]^.

 In salivary gland pathology, mucocele was the most frequently reported lesion detected in lower lip. The lesion was found predominantly in females. Other authors have stated that mucocele is the most common oral lesion in children^[^^6^^,^^10^^,^^11^^,^^13^^,^^15^^,^^17^^,^^20^^]^, but its incidence was lower in our study. Also, Shulma^[^^21^^]^ in a large group of patients under 17 years found only 5 cases of mucocele. This lesion almost equally affected the males and females, but Nico et al have found a female predilection^[^^22^^]^.

 In the group of pulp and periapical lesions, similar to Maya et al, radicular cyst was the most common lesion which involved maxillary bone more frequently than mandible^[^^17^^]^. Many authors have included this cyst in odontogenic cysts group and have reported this lesion as the second common cyst after dentigerous cyst^[^^23^^]^. 

 Within odontogenic tumors, odontoma and ameloblastoma were the most frequently found hamartoma and tumor in maxilla and mandible respectively. It is in agreement with other studies that have reported these lesions as first and second common odontogenic tumor^[^^7^^,^^10^^,^^11^^,^^17^^,^^20^^]^. Maya et al have found odontoma in mandible more frequently^[^^17^^]^. The male to female ratio for odontoma and ameloblastoma was 2:1 and 1.25:1 respectively, which is comparable with other studies^[^^6^^,^^12^^,^^24^^]^. However, there are some reports that have demonstrated female or male predilection or an equal gender distribution for both lesions.

 Although in the new classification of World Health Organization (WHO), OKC have been defined as an odontogenic tumor^[^^8^^]^ and authors who have considered this classification, have reported keratocystic odontogenic tumor (KOT) as the most common odontogenic tumor^[^^4^^,^^6^^,^^10^^,^^11^^,^^13^^,^^17^^, ^^20^^]^. Regarding the anatomic location, we found predominance of maxillary bone. Authors in Africa^[^^12^^]^, Thailand^[^^7^^]^ and Nigeria[^24^^]^ have reported results which are in line with our findings, but Latrou et al^[^^16^^]^, studied odotomas in Greece population and have noted that mandible and maxilla have been involved equally. 

 We found only 13 cases of epithelial lesions. Gingiva was the most frequently affected site. Epithelial hyperplasia and squamous papilloma were the most common lesions in this category. Unfortunately, there are not many available researches about these lesions. Wang et al^[13]^ have demonstrated only a few cases of epithelial hyperplasia. These lesions showed female predilection in our study which is in agreement with a study in UK^[^^10^^]^ but Wang et al have found male predominance in Taiwan^[^^13^^]^. However, due to the limited number of recorded cases, this difference is acceptable. 

 The incidence of hematopoietic disorders was 1.2%, this included 5 cases of Burkitt's lymphoma and 2 cases of eosinophilic granuloma. In other studies this incidence was lower than 5%^[^^6^^,^^10^^,^^17^^,^^20^^]^. But in Lowoyin et al’s study the prevalence was 11%^[^^12^^]^. This high incidence is due to the endemic occurrence of Burkitt's lymphoma in African children. Out of five cases of Burkitt’s lymphoma, 4 cases occurred in males. This male predilection is similar to other studies^[^^6^^,^^12^^,^^20^^]^. 

 In this study we found only 0.86% malignant neoplasms including 5 cases of Burkitt’s lymphoma, 2 cases of mucoepidermoid carcinoma and one case of adenoid cystic carcinoma, with a M:F ratio of 0.7:1. The incidence of malignant lesions was similar to the previous studies in Taiwan^[^^6^^]^, UK^[^^10^^]^ and Thailand^[^^7^^]^. However, the studies on African populations have demonstrated a high rate of malignancy; most of them were lymphomas^[^^12^^,^^13^^]^. These results might be explained by some deficiencies and genetic susceptibilities in African children^[^^12^^,^^13^^]^.

## Conclusion

Our results revealed that nearly 19.2% of lesions occurred in patients less than 18 years with an equal gender distribution. The majority of lesions were soft tissue diseases with a reactive nature. However, there were some differences in comparison with other studies; for instance, our rate was higher in soft tissue lesions and a lower prevalence of mucocele. These data could help the dentists and surgeons for more accurate management of their patients.

 Similar studies in various groups of patients should be designed to obtain an actual prevalence and accurate demographic data for any oral disease.

## Authors’ Contribution

Z. Jaafari: conceived and designed the study and prepared the manuscript. 

Z. Ahmadi: performed the literature search,collected data, initially analyzed and interpreted the data. 

F. Kamali: contributed in writing of initial manuscript. 

All authors read and approved the final version of the manuscript.
